# Interferon-Gamma Release Assay Performance of Pleural Fluid and Peripheral Blood in Pleural Tuberculosis

**DOI:** 10.1371/journal.pone.0083857

**Published:** 2013-12-27

**Authors:** Fei Liu, Mengqiu Gao, Xia Zhang, Fengjiao Du, Hongyan Jia, Xinting Yang, Zitong Wang, Liqun Zhang, Liping Ma, Xiaoguang Wu, Li Xie, Zongde Zhang

**Affiliations:** 1 Beijing Key Laboratory for Drug Resistant Tuberculosis Research, Beijing Chest Hospital, Capital Medical University, Beijing Tuberculosis and Thoracic Tumor Research Institute, Beijing, China; 2 Tuberculosis Department, Beijing Chest Hospital, Capital Medical University, Beijing Tuberculosis and Thoracic Tumor Research Institute, Beijing, China; 3 Thoracic Surgery Department, Beijing Chest Hospital, Capital Medical University, Beijing Tuberculosis and Thoracic Tumor Research Institute, Beijing, China; Institute of Infectious Diseases and Molecular Medicine, South Africa

## Abstract

**Background:**

The diagnosis of pleural tuberculosis (TB) remains to be difficult. Interferon-gamma release assay (IGRA) is a promising method for diagnosing TB in low TB burden countries. The release of interferon-gamma (IFN-γ) by T lymphocytes increases at a localized site of infection with *Mycobacterium tuberculosis* antigen. This study aimed to examine the clinical accuracy of T-SPOT.TB on pleural fluid and peripheral blood for the diagnosis of pleural TB in high TB burden country.

**Methods:**

168 subjects with pleural effusion were enrolled prospectively and examined with T-SPOT.TB on pleural fluid and peripheral blood samples simultaneously.

**Results:**

The receiver operating characteristic (ROC) curve and cut-off value of pleural fluid T-SPOT.TB was established according to spot forming cells (SFC) between culture/biopsy-confirmed pleural TB group and no pleural TB group. The sensitivity of pleural fluid T-SPOT.TB and peripheral blood T-SPOT.TB was similar (96.3% and 92.7%, respectively) (P= 0.691). In contrast, the specificity of pleural fluid T-SPOT.TB (94.5%) was significantly higher than that of peripheral blood T-SPOT.TB (76.1%) (P=0.002). 2% (2/98) of pleural fluid T-SPOT.TB results were indeterminate.

**Conclusion:**

The diagnostic accuracy of peripheral blood T-SPOT.TB is low in high TB burden countries due to latent tuberculosis infection. Pleural fluid T-SPOT.TB is a relatively useful and supplementary test to explore pleural TB in high TB burden countries, but its diagnostic accuracy needs to be validated in further large scale research.

## Introduction

 Tuberculosis (TB) is the leading cause of death from a curable infectious disease. Eight to ten million of new TB cases are reported each year in high burden developing countries (Who, 2011). In China, 4.99 millions of new cases of active TB (ATB) are reported each year according to the fifth national-wide TB survey in 2010. TB is the major cause of pleural fluids (PF) in areas of high TB prevalence, and may account for 25% of all pleural effusions [1]. Annually, over half a million of cases of pleural effusion resulting from TB occur worldwide [2]. Early and accurate diagnosis of pleural TB remains to be a problem due to the difficulties in differential diagnosis between pleural TB and malignant pleural effusion. Misdiagnosis of pleural TB leads to inappropriate treatment of patients, causing unnecessary suffering to the patient. Although bacteria culture and histology are the gold standard of diagnosis for pleural TB, low abundance of bacteria in PFs results in insensitivity of Ziehl-Neelsen staining and bacteria culture [3,4]. Conventional biochemical and cellular characterization of PF lacks specificity [5]. Currently, biopsy of pleural tissue is widely held to be the direct method for confirming the diagnosis. However, pleural biopsy is invasive, operator-dependent and technically difficult (particularly in children)[6]. Up to date, adenosine deaminase (ADA), an enzyme associated with T-lymphocyte activity, is the most cost-effective PF marker and is routinely used in high prevalence settings. However, some researches indicated that the sensitivity of ADA was lower for the diagnosis of pleural TB. ADA increases can also be observed in other types of infection, malignant diseases and rheumatic diseases. and lympho-proliferative disorders, and thus is not specific for pleural TB[7,8]. Therefore, a rapid, accurate diagnostic test is urgently needed for pleural tuberculosis.

 Recently, the interferon-γ release assays (IGRAs) are being used increasingly to detect IFN-γ response to the Mycobacterium tuberculosis-specific antigens, early secretory antigenic target 6 (ESAT-6) and culture filtrate protein 10 (CFP-10). Genes encoding these antigens are present in Mycobacterium tuberculosis, but absent from BCG strain and most environmental non-tuberculosis mycobacteria (NTM) strains [9]. In low TB burden countries, IGRAs have been considered to be alternative useful tools for the diagnosis of active TB . However, in high TB burden countries, cases of latent tuberculosis infection (LTBI) are highly abundant that will inevitably affect the diagnostic accuracy of peripheral Blood (PB) IGRAs. Thus, this assay is questionable as a diagnostic marker in high TB burden countries [10,11].

 During active TB, mycobacterium-specific T cells proliferate and are recruited to the site of infection where the number of effector T cells is much higher than those in PB [12,13]. Jafari et al. demonstrated that active pulmonary TB can be confirmed rapidly with an ELISPOT assay in bronchoalveolar lavage fluid in smear-negative setting, while another study on 36 patients with PF in an area with intermediate TB burden country (South Korea) suggested the PF T-SPOT.TB could be the most useful test among the interferon-gamma releasing assays [14,15]. Whether PB T-SPOT.TB and PF T-SPOT.TB can be useful tools for diagnosing pleural TB in high TB burden countries remains unclear.

 In this study, we investigated ELISPOT based IGRAs (T-SPOT.TB) applied to both PF and PB obtained from patients with pleural effusion in order to evaluate the diagnostic performance of this assay for the diagnosis of pleural TB in China. 

## Methods

 This study received ethical approval from the Ethics Committee of the Beijing Chest Hospital, Capital Medical University. Informed consent was obtained from all participants in the written form.

### Study participants

**Figure 1:**
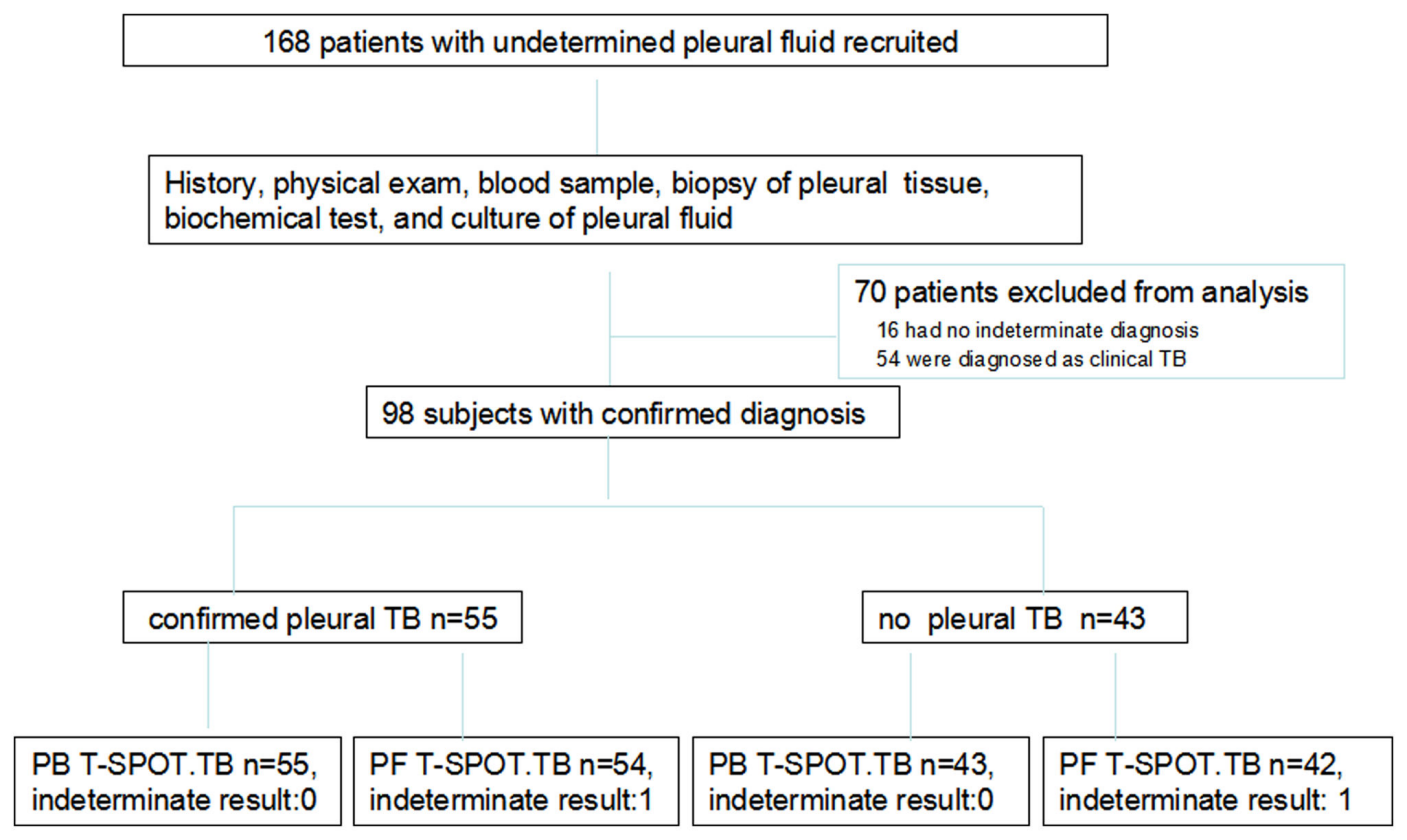
Flowchart of the study population. A total of 168 patients with undetermined PF were recruited, 98 were eligible to be included in the final analysis (these patients had paired PB T-SPOT.TB and PF T-SPOT.TB). TB, pleural tuberculosis; no TB, no pleural tuberculosis (final diagnosis excluded pleural tuberculosis).

 This prospective study was conducted in Beijing Chest Hospital. A total of 168 patients with pleural effusion of undetermined etiology were enrolled from May 2012 to June 2013. Patients were tested with PB T-SPOT.TB and PF T-SPOT.TB at enrollment. Medical records were collected on age, gender, underlying disease, BCG scar and HIV serology status results. Routine clinical, microbiologic, histopathological and biochemical examinations of PFs and sputum samples were also performed. Individuals were excluded if they have previous tuberculosis history & tuberculosis contact history or they have received anti-tuberculosis therapies before enrollment. Major clinical characteristics of recruited subjects were summarized in Table 1. Altogether, 70 patients were excluded from the study due to no confirmed diagnosis, among which 58 patients had clinical TB without culture or biopsy evidences. (Figure 1).

**Table 1:** Clinical characteristics and biochemical characteristics of PF in study population (n=98).

**Characteristics**	**Pleural TB group (n=55)**	**No pleural TB Group (n=43)**	P Value
Age, median (range), yrs	39(25-59,)	57(47-67)	0.003
Gender(female/male,)	18/37	18/25	0.200
BCG scar	5	4	1.000
Complications			
Diabetes mellitus	9	7	0.991
ADA, U/L	48.15(35.63-66.43)	12.35(8.9-16.73)	0.000
Lymphocyte, /μl	2124(1285-3342)	965(615.25-2153.5)	0.018
Rivalta test^*^, (n)	36/9	37/5	0.305
Albumin, mg/dl	47.20(41.40-52.45)	46.10(37.43-51.93)-	0.165
LDH, IU/L	282.4(186.6-565.2)	367.10(261.38-584.25)	0.912
CRP, mg/ml	16.04(5.41-26.63)	4.79(2.24-17.40)	0.018
Cause of effusion	Microbiology (n=28)	Malignant PF (n=35)	
	Histopathology (n=27)	Parapneumonia effusion (n=4)	
		Autoimmune disease (n=3)	
		pulmonary embolism(n=1)	

Abbreviations: TB, tuberculosis; ADA, adenosine deaminase; LDH, lactate dehydrogenase; CRP, C reactive protein.

^*^ Rivalta test: is used as a puncture fluid test for differentiation of exudate and transudate.

### Definitions and Diagnosis

 The final diagnosis was based on clinical manifestations, bacteriological, biochemical examinations, histopathological examination. Finally, all of the patients are diagnosed as either (1) culture/biopsy-confirmed pleural TB: if final diagnoses were made on the positive culture of M. TB from PF or the presence of caseating granuloma in pleural biopsy specimen; or (2) no pleural TB: other diagnoses except tuberculosis were made by microbiologic, histopathological, and serologic examinations. 

### T-SPOT.TB assay

 The T-SPOT.TB test (Oxford Immunotec Ltd., Abingdon, UK) was performed according to the manufacturer's instructions. 6 ml of heparinized PB sample and 50 ml of PF samples were collected within 2 h after the collection. For the T-SPOT.TB assay of PF, the specimens were centrifuged at 4,000 rpm for 10 min. The supernatants were discarded and the sediments were resuspended in 5ml phosphate buffer, the subsequent processes were the same as for the test using blood samples. PF mononuclear cells (PFMC) and PB mononuclear cells (PBMC) were isolated and adjusted to concentration of 2.5×10^6^/ml and then inoculated with two antigens (ESAT-6 and CFP-10). The procedure was performed in the plates pre-coated with anti-interferon-γ antibodies at 37°C for 16 to 20 hours. After application of alkaline phosphatase-conjugated second antibody and chromogenic substrate, the number of Spot Forming Cells (SFCs) in each well was automatically counted with a CTL ELISPOT system (CTL-ImmunoSpot® S5 Versa Analyzer, USA). 

**Figure 2:**
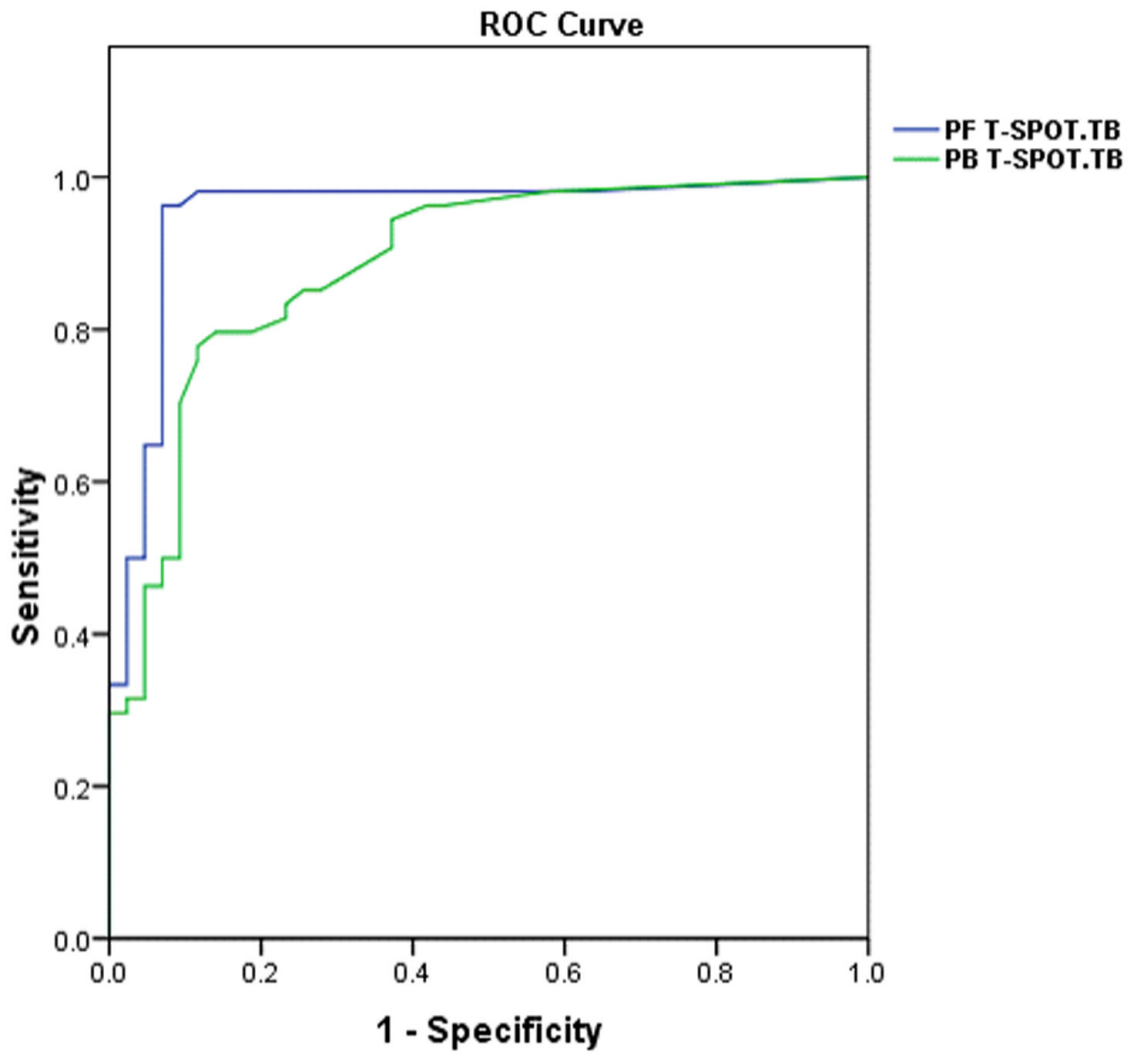
Receiver operating characteristic curve in PF T-SPOT.TB and PB for the diagnosis of pleural tuberculosis. The horizontal line in scatter plot stands for median of spot forming cells. SFCs, spot forming cells; TB, pleural tuberculosis; no TB, no pleural tuberculosis.

 We derived new cut-off value of PF using receiver operating characteristic curve (ROC) analysis according to SFCs between culture/biopsy-confirmed pleural TB group and no pleural TB group (Figure 2). PF T-SPOT.TB results were considered positive if more than 54 spot forming cells (SFC) were counted after subtraction of the number of SFCs in the negative control well and if the total number of SFCs was at least twice the number of SFCs in the negative control well. Results were considered negative if they did not meet the definition for a positive result, that was if less than 54 SFCs were counted after subtraction of the number of SFCs in the negative control well or if the total number of SFCs was less than twice the number of SFCs in the negative control well. Results were determined to be indeterminate (1) if the positive control failed (2), if there was high background discoloration in the wells precluding meaningful evaluation of the plate.

 The diagnostic criteria of PB T-SPOT.TB for positive, negative and indeterminate outcome was recommended by the manufacturer.

### Mycobacterial culture and strain identification of PF

 Specimen processing: All specimens for diagnosis of pleural tuberculosis were processed using standard techniques and procedures according to Chinese Anti-tuberculosis Association Guidelines [16]. Specimens with solid pleural tissue were homogenized and then decontaminated with 4% NaOH for 10min. 50ml PF specimens were centrifuged at 4,000 rpm for 10 min. The supernatants were discarded and the sediments were resuspended in 2ml phosphate buffer. Mycobacterial cultures were conducted by inoculating resulting suspension and the processed solid pleural tissue of the specimens onto solid L-J media (Lownstein-Jenson, L-J). If mycobacterium culture is proved to be positive, then strain identification was needed to identify Mycobacterium tuberculosis (M.TB).

### Thoracoscopic pleural biopsy

 Pleural biopsy was performed if possible, pleural tissue was submitted to histopathological study and acid-fast bacterium(AFB) staining. The diagnosis of confirmed pleural tuberculosis was made if specimens showed caseating granuloma.

### Statistical analyses

 Continuous variables were compared using nonparametric Mann-Whitney U test. A ROC curve was constructed by plotting the rate of sensitivity against the rate of (1-specificity) results over a range of cut-off values of PF T-SPOT.TB and PB T-SPOT.TB. Youden's Index was used to select the optimum cut points on the ROC curve (optimal balance between sensitivity and specificity). Comparisons between proportions were performed using Chi-squared test. Ninety-five percent confidence intervals (95%CI) were estimated according to the binomial distribution. Significance was inferred for P<0.05. All statistical analysis was performed using the commercial statistical software SPSS version 14.0 (SPSS, Inc., Chicago, IL, USA).

## Results

### Clinical characteristics

 Among the 168 patients with PF of undetermined etiology, 55 were diagnosed as culture/biopsy confirmed pleural TB and 43 were diagnosed as no pleural TB. Clinical characteristics of these 98 patients are shown in Table 1. Average age of no pleural TB group (57) was higher than that of TB group (39). There were 5 and 4 cases without BCG scar in pleural TB group and no pleural TB group respectively, and these patients were all from remote rural area. All patients in the study were negative in HIV serology test. The main complication was diabetes mellitus, but there was no significant difference in pleural TB group and no pleural TB group. Lymphocyte count, adenosine deaminase (ADA) activity and C reactive protein (CRP) of PF in pleural TB group were higher than those in no pleural TB group. TB distribution of affected organs other than pleural tissue was highly heterogeneous including lung, lymph node, bronchus, bone, intestine and peritoneal TB (Table 2). 

**Table 2:** Pleural TB complicated with other organ TB.

Other organ TB	Cases (n=38)
Pulmonary TB	30
Pulmonary &Intestinal TB	1
Lymph node TB	4
tuberculous peritonitis	2
Osteoarticular TB	1

### Establishment of Receiver operating characteristic (ROC) curve of PF and PB

 Compared to PB, distinct cut points in specific body compartments have been used. However, no cut-off for PF, which has a unique physiologic and anatomic characteristics, has been defined. We established a new ROC curve of PF and defined 216 spot forming cells (SFCs) per million PFMC as the optimal cut-off value that could maximize specificity without a significant loss of sensitivity. Area under curve (AUC) of PF T-SPOT.TB and PB T-SPOT.TB were 0.950 (95% CI, 0.901-0.999) and 0.885 (95% CI, 0.818-0.952), respectively . 

### Diagnostic performance of T-SPOT.TB in PF and PB

**Figure 3:**
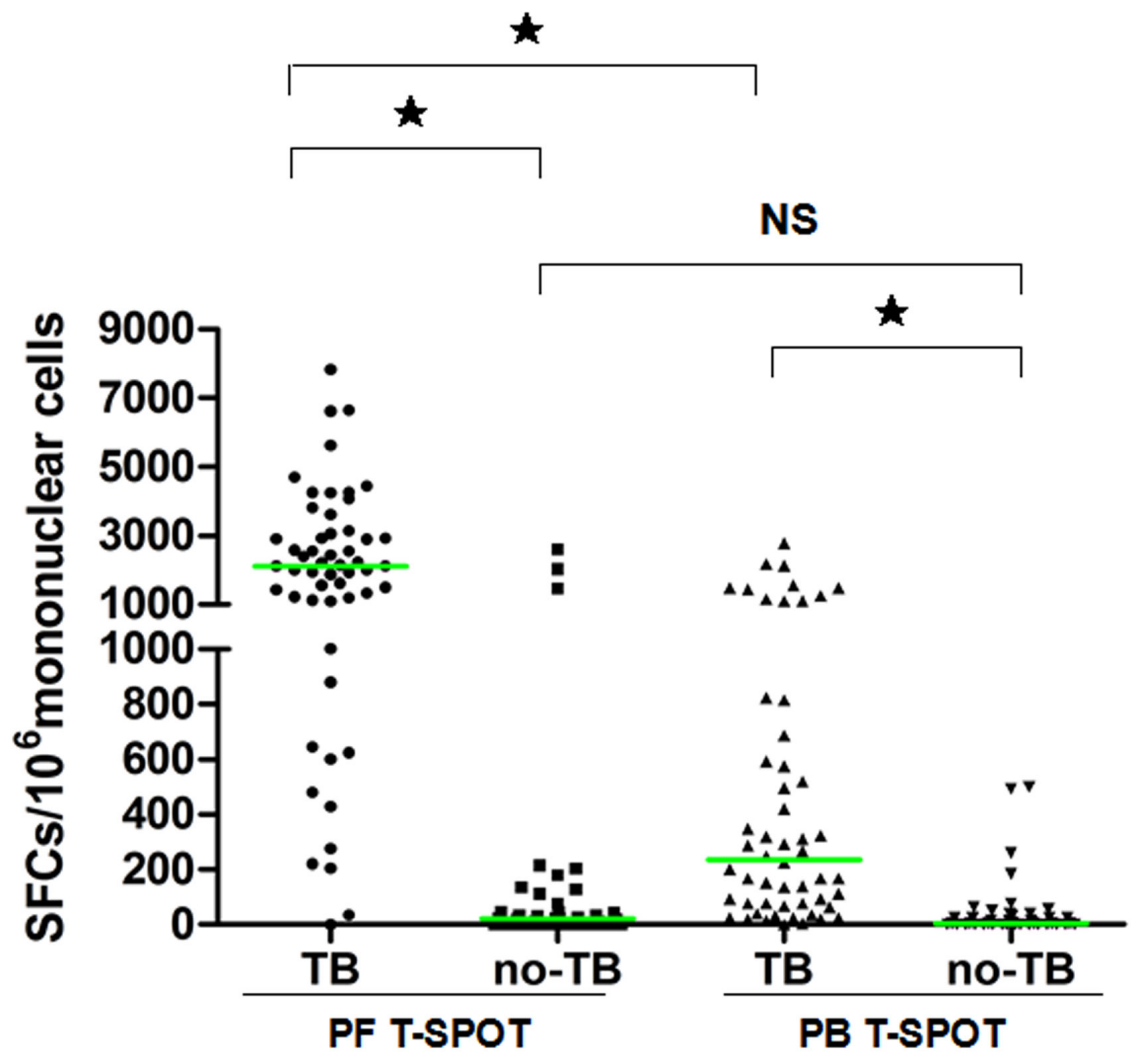
Scatter plot of spot forming cells using PF T-SPOT.TB and PB T-SPOT.TB between pleural tuberculosis and no pleural tuberculosis. For group comparison by Mann-Whitney Test. ★P<0.0001; NS, not significant.

 Compared to the no pleural TB group, the pleural TB group had significantly more SFCs in PFMC and PBMC. Within the pleural TB group, there were more SFCs in PFMC than those in PBMC. However, within the no pleural TB group, there was no significant difference in SFCs between PF and PB (Figure 3).

 The diagnostic performance of PF T-SPOT.TB and PB T-SPOT.TB for 98 subjects was presented in Table 2. The overall sensitivity of PF T-SPOT.TB and PB T-SPOT.TB was 96.3% (95% CI, 84.5%-99.0%) and 92.7% (95% CI, 82.7%-97.1%), demonstrating that there was no difference in sensitivity between these two tests. In marked contrast, the specificity of PF T-SPOT.TB (92.9%; 95% CI, 81.0%-97.5%) is significantly higher than that of PB T-SPOT.TB (62.8%; 95%CI, 47.9%-76.0%). PPV, NPV, LR+ and LR- of PF T-SPOT.TB were 94.5%, 95.1%, 13.481 and 0.040, and the corresponding parameters of PB T-SPOT.TB were 76.1%, 87.1%, 2.492 and 0.116 (Table 3).

**Table 3:** Diagnostic performance of PF T-SPOT.TB and PB T-SPOT.TB in study population (n=98).

	**PB T-SPOT.TB**		**PF T-SPOT.TB**	
**parameter**	**Value**	**95%CI**	**Value**	**95%CI**
Sensitivity,%(n)	92.7(51/55)**^*a*^**	82.7-97.1	96.3(52/54 )^*^	84.5-99.0
Specificity,%(n)	62.8(27/43)**^*b*^**	47.9-76.0	92.9(39/42) ^*^	81.0-97.5
PPV,%(n)	76.1(51/67)	64.7-84.7	94.5(52/55)	85.2-98.1
NPV,%(n)	87.1 (27/31)	71.2-94.9	95.1(39/41)	83.9-98.7
LR+	2.492	1.678-3.700	13.481	4.525-40.165
LR-	0.116	0.044-0.306	0.040	0.010-0.156
AUC	0.885	0.818-0.952	0.950	0.901-0.999

Abbreviations: PPV, positive predictive value; NPV, negative predictive value; LR+, likelihood ratio for positive test; LR-, likelihood ratio for negative value.

^*^there were 1 indeterminate PF T-SPOT.TB results in pleural tuberculosis group and no pleural TB group respectively; ***^a^*** P=0.691, comparison of PB T-SPOT.TB and PF T-SPOT.TB in pleural tuberculosis group; ***^b^*** P=0.002, comparison o f PB T-SPOT.TB and PF T-SPOT.TB in no pleural TB group.

### Indeterminate results in PF T-SPOT.TB

 In terms of PF T-SPOT.TB results, indeterminate rate was 2.0% (2/98; 95%CI, 0.6%-7.1%). There was 1 indeterminate result in pleural TB and no pleural TB group, respectively. All indeterminate results were due to high background discoloration within wells, which precluded spot visualization. There was no indeterminate result in PB T-SPOT.TB. 

### Comparison of PF ADA and PF T-SPOT.TB

 We also compared the sensitivity and specificity of PF T-SPOT.TB with those of PF ADA. We found that although there was no significant difference in specificity of PF ADA (93%; 40/43) and (92.9%; 39/42). However, the sensitivity (96.3%; 52/54) of PF T-SPOT.TB (72.7%; 40/55) is significant higher than that of PF ADA. 

## Discussion

 In this study, we prospectively investigated possible clinical utilization of the T-SPOT.TB assay applied to PF and PB for the diagnosis of pleural TB in high TB burden area. Our data indicate that the PF T-SPOT.TB, rather than the PB T-SPOT.TB, is a rapid and accurate assay for diagnosis of pleural TB in high burden TB countries. 

 Our study initially recruited 168 patients with pleural illness, routinely seen by clinicians in a high-burden setting. Subsequently, patients clinical diagnosed as pleural TB, but not confirmed by bacterial culture and pathology, were excluded from analysis since it is possible that the clinical TB feathers are caused by some other diseases such as pleural mesothelioma and parapneumonic effusions. Inclusion of these subjects might possibly bias the diagnostic performance of T-SPOT.TB. Nevertheless, we did not detect significant difference in both sensitivity and specificity of PF-SPOT. TB between clinically diagnosed pleural TB group and no TB group (data not shown). To our knowledge, up to date, this is the largest study for evaluation of the diagnostic performance of IGRAs using consecutively recruited patients with culture/biopsy-confirmed pleural TB.

 In our study, the sensitivity of PB T-SPOT.TB for detecting pleural TB was consistent with previous studies conducted in low and intermediate TB burden countries [17,18,19]. However, the specificity of our PB T-SPOT.TB for detecting pleural TB was lower than that in studies conducted in low and intermediate TB burden countries in which a specificity of 67.0%-90.5% was reported [15,18,19]. The main reason for the lower specificity of PB in our study might be due to the high LTBI in the control group. The positive rate of PB T-SPOT.TB in the control group (LTBI rate) was 37.2% (16/43), which is consistent with the LTBI rate (44.5%) in a national-wide TB survey in 2000 in China [20,21,22]. To date, IGRA is the best promising test for the diagnosis of LTBI, especially in the BCG-vaccinated populations. However, no baseline survey has been carried out in China for the prevalence of LTBI determined by T-SPOT.TB to date. 

 The sensitivity of PF T-SPOT.TB was similar to that of PB T-SPOT.TB in our study. However, the specificity of PF T-SPOT.TB PF was significantly higher than that of PB T-SPOT.TB. Recently, high specificity of PF T-SPOT.TB was also reported in several studies carried out in intermediate TB burden countries such as Taiwan and South Korea [15,19]. The low specificity of PB T-SPOT.TB was possibly becuase that the immune responses assayed on PBMC could only provide background information about effector T cell activity in active TB. Only a minority of the lymphocytes in the human body is found in PB, and the frequency with which MTB specific T-cells occur in PB is very low, even in ATB [14]. Therefore, it has been impossible to discriminate between active TB and LTBI using these assays. The relatively high specificity of PF T-SPOT. TB for pleural TB was possibly due to compartmentalization of antigen-specific effector T cells. Effector T cells could be recruited and expanded among lymphocytes from localized tuberculosis infection site, such as pleural cavity. Consistent with this possibility, Wilkinson et al. reported that ESAT-6-specific, IFN-γ secreting T-cells were concentrated 15-fold in PF relative to their blood level in patients with known pleural TB [23]. Therefore, we postulated that effector T cells in PF could be less affected than PB by LTBI. Thus, IGRA responses detected in PFMC could provide relatively better discrimination of ATB from LTBI than responses to PBMC alone [24]. 

 In the current study, there were 2 indeterminate results in PF T-SPOT.TB due to high background discoloration within wells that precluded spot visualization. Tuberculous pleural effusion usually contains a large quantity of cells, protein, blood plot and fibrin that may cause the high background discoloration. Consistent with this possibility, there were no indeterminate results in PB T-SPOT.TB. For some unknown reasons, our results are inconsistent with a previous report, in which the pooled indeterminate rate of PB T-SPOT.TB was 8.2% and the indeterminate results were thought to be due to different level of IFN-γ production [25]. 

 In our study, we found that although there was no difference in specificity between PF ADA and PF T-SPOT.TB, the sensitivity of PF ADA is significantly lower than that of PF T-SPOT.TB. There are two possible reasons may cause the relatively low sensitivity of ADA. First, it has been shown that some patients in the early phase of pleural TB have low PF ADA levels. Second, ADA has been suggested to be a less sensitive marker of pleural TB in immunocompromised patients [26]. A variety of pleural conditions lead to an elevated PF ADA level, with pleural malignancies (especially lymphomas), rheumatoid pleurisy and parapneumonic effusion being the leading causes. In our study, among the 3 ADA positive cases in no pleural TB group, 1 subject is suffered from parapneumonic pleural effusion and two other cases are suffered from lymphoma[27]. 

 The limitations of our study need to be addressed. First, the number of cases was small, the confidence intervals of our test outcomes could have been narrower if we had recruited a larger cohort. Second, establishment of ROC curve and the cut-off value relied on SFCs of PF and PB in culture/biopsy-confirmed pleural TB and no pleural TB group. Thus, subjects with LTBI in no pleural TB group would influence the accuracy. However, these cannot be absolutely avoided by careful inquiry and examination due to the high prevalence of LTBI in China. Third, we get the optimal cut-off of PF T-SPOT.TB from ROC analysis in this training cohort, and the definite accurary of PF T-SPOT.TB for the diagnosis of pleural TB need the further validation cohort. 

 Our study suggests that the diagnostic accuracy of PF T-SPOT.TB might be higher than that of PB T-SPOT.TB by comparing the cut-offs for PF T-SPOT.TB and PB TSPOT.TB. However, as the cut-off of PF T-SPOT.TB was derived from ROC analysis of this cohort, whereas that of PB TSPOT.TB has been validated, the definite diagnostic value of PF T-SPOT.TB should be proved by further large scale validation cohort [28]. 
